# Trends in Hospital Resource Use for Children With Complex Chronic Conditions

**DOI:** 10.1001/jamanetworkopen.2025.44686

**Published:** 2025-12-02

**Authors:** Nathaniel D. Bayer, Matthew Hall, Maria Osipovich, John M. Morrison, Christian D. Pulcini, Jana C. Leary, Joanna E. Thomson, Tamara D. Simon, Dennis Z. Kuo, Jeffrey D. Colvin, Eyal Cohen, Jay G. Berry

**Affiliations:** 1 Division of Pediatric Hospital Medicine, Department of Pediatrics, Golisano Children’s Hospital, University of Rochester, Rochester, New York; 2 Children’s Hospital Association, Lenexa, Kansas; 3 Division of General Pediatrics, Department of Medicine, Boston Children’s Hospital, Harvard Medical School, Boston, Massachusetts; 4 Department of Pediatrics, John Hopkins All Children’s Hospital, Saint Petersburg, Florida; 5 Department of Emergency Medicine, Larner College of Medicine, University of Vermont, Burlington; 6 Department of Pediatrics, Larner College of Medicine, University of Vermont, Burlington; 7 Division of Hospital Medicine, Cincinnati Children’s Hospital Medical Center, Cincinnati, Ohio; 8 Department of Pediatrics, University of Cincinnati, Cincinnati, Ohio; 9 Department of Pediatrics, Keck School of Medicine, University of Southern California Los Angeles; 10 Division of Developmental and Behavioral Pediatrics, Department of Pediatrics, Golisano Children’s Hospital, University of Rochester, Rochester, New York; 11 Department of Pediatrics, Children’s Mercy Hospital, Kansas City, Missouri; 12 Department of Pediatrics, Hospital for Sick Children, University of Toronto, Toronto, Ontario, Canada

## Abstract

**Importance:**

Despite a small prevalence, children with complex chronic conditions (CCCs) use substantial inpatient resources.

**Objective:**

To assess national trends in hospital discharges, bed days, and hospital charges for children with and without CCCs in the US from 2000 to 2022.

**Design, Setting, and Participants:**

This retrospective, repeated cross-sectional study used hospital discharge data from the Kids’ Inpatient Database (KID) from the years 2000, 2003, 2006, 2009, 2012, 2016, 2019, and 2022 for US children aged 0 to 18 years, excluding uncomplicated newborn discharges.

**Exposure:**

Presence of 0, 1, 2, or 3 or more CCCs.

**Main Outcomes and Measures:**

Trends in the hospital discharge rate per 100 000 children and percentage of total hospital discharges, bed days, and charges attributable to children with CCCs, identified with *International Classification of Diseases, 9th Revision, Clinical Modification* and *International Statistical Classification of Diseases and Related Health Problems, 10th Revision, Clinical Modification* codes using Feudtner’s diagnosis code classification system, version 3. Survey weights were applied to estimate hospital discharges, bed days, and charges. Sociodemographic (eg, primary payer) and clinical (eg, technology dependence, mental health comorbidity) characteristics for each hospital discharge were also assessed.

**Results:**

Across all years, there were an estimated 26 342 497 hospital discharges, of which 54.1% (95% CI, 54.0%-54.2%) were among males and 55.4% (95% CI, 54.4%-55.8%) were for infants. From 2000 to 2022, the discharge rate per 100 000 US children increased by 24.3% (95% CI, 22.7%-26.3%), from 779 to 968, for children with 1 or more CCCs and decreased by 9.7% (95% CI, 9.4%-10.0%), from 3831 to 3459, for children with no CCCs. From 2000 to 2022, the percentage change in the hospital discharge rate varied by number of CCCs: a 3.8% (95% CI, 0.9%-6.0%) decrease was found for 1 CCC, a 60.9% (95% CI, 57.7%-65.5%) increase for 2 CCCs, and a 340.0% (95% CI, 332.6%-351.1%) increase for 3 or more CCCs. In 2000 and 2022, children with 1 or more CCCs accounted for 16.9% (95% CI, 15.7%-17.9%) and 21.9% (95% CI, 20.7%-22.9%) of hospital discharges, 32.0% (95% CI, 30.8%-33.1%) and 44.1% (95% CI, 42.6%-45.4%) of bed days, and 44.2% (95% CI, 42.6%-45.5%) and 59.5% (95% CI, 57.8%-60.9%) of hospital charges, respectively. From 2000 to 2022, the percentage of hospital discharges in children with 1 or more CCCs increased with gastroenterologic technology dependence (7.0% [95% CI, 6.0%-8.0%] to 14.4% [95% CI, 12.4%-16.4%]), neurodevelopmental or neurocognitive disorders (5.7% [95% CI, 4.8%-6.5%] to 13.5% [95% CI, 11.7%-15.2%]), and public insurance (40.9% [95% CI, 38.8%-42.9%] to 52.1% [95% CI, 50.2%-54.1%]).

**Conclusions and Relevance:**

In this national, repeated cross-sectional study, the hospital discharge rate and the percentage of hospital resource use attributable to children with CCCs increased from 2000 to 2022, and these trends were mainly attributable to children with multiple CCCs. It is critical that health systems are equipped with the resources, staff, and payments to sustainably meet the increasing needs for inpatient care among children with CCCs.

## Key Points

**Question:**

What were the trends in hospital discharges and hospital resource use for US children with complex chronic conditions (CCCs) from 2000 to 2022?

**Findings:**

In this cross-sectional study of 26 342 497 pediatric discharges from the Kids’ Inpatient Database from 2000 to 2022, significant increases occurred in the hospital discharge rate and the percentage of total discharges, bed days, and hospital charges attributable to children with CCCs, especially multiple CCCs.

**Meaning:**

The findings suggest the case mix of pediatric hospitalizations has become increasingly complex, emphasizing the need for health systems, policymakers, training programs, and payers to optimize inpatient care for children with CCCs.

## Teaser

This cross-sectional study assesses trends in hospital discharges and use of hospital resources from 2000 to 2022 by US children aged 0 to 18 years who have complex chronic conditions.

## Introduction

Pediatric complex chronic conditions (CCCs) are empirically aggregated from mortality risk and are defined as conditions lasting more than 12 months, requiring specialty care, and often necessitating hospitalization.^1^ Hospitalizations account for nearly one-half of health care spending on children with CCCs.^2^ Multimorbidity is common among hospitalized children with CCCs and can manifest with co-occurring technology dependence (eg, gastrostomy), mental health diagnoses (eg, anxiety),^3^ and other multiple CCCs. Multimorbidity in children is associated with inpatient medical errors,^4^ postoperative complications,^5^ prolonged length of stay,^6^ insufficient postdischarge supports,^7^ and hospital readmission.^8,9^

The prevalence of children with multiple CCCs is small (<1% of all children) but increasing.^10^ Studies from prior decades on hospitalized children with multiple CCCs report increases in discharge rates,^11^ hospital bed days and charges, reliance on Medicaid to cover hospital care,^2,12^ and co-occurring mental health conditions.^12,13^ Findings from recent studies suggest this impact has continued, especially in US children’s hospitals.^14,15^ Existing studies on trends in hospitalizations for children with CCCs did not account for policies and practices that resulted in the increase of diagnosis codes submitted with hospitals’ administrative billing claims, which could have affected the results.^16,17^

Therefore, we assessed US population trends in hospitalizations for children with CCCs from 2000 to 2022, including (1) hospital discharge rates, bed days, and charges and (2) technology dependencies and mental health conditions. We incorporated adjustment for diagnosis coding intensity. The findings from this work will inform health systems’ planning for anticipated CCC hospitalizations and may help direct hospital clinicians’ efforts to deliver inpatient care to children with CCCs.

## Methods

### Study Design, Data Source, and Population

We performed a retrospective repeated cross-sectional study of hospital discharges for children in the Agency for Healthcare Research and Quality’s (AHRQ) Kids’ Inpatient Database (KID).^18^ KID is the largest publicly available all-payer pediatric inpatient database in the US. Managed through the AHRQ’s Healthcare Cost and Utilization Project (HCUP),^19^ KID contains deidentified sociodemographic, clinical, and hospital data for each inpatient discharge. KID does not include discharges from observation-level care, which refers to short-term assessment and treatment intended to determine whether a patient can be discharged or requires inpatient admission.^20,21^ The institutional review board at the University of Rochester deemed the study not human participants research and exempted it from approval and informed consent. This study followed the Strengthening the Reporting of Observational Studies in Epidemiology (STROBE) reporting guideline.^22^

We analyzed hospital discharges for children aged 0 to 18 years excluding uncomplicated newborn discharges, defined in KID as an in-hospital birth with the diagnostic-related group “normal newborn” (eTable 1 in Supplement 1). We retained complicated newborn discharges because some of these newborns could have a CCC. Each KID includes a full calendar year of hospital discharges. From 2000 to 2022, 8 KIDs are available: 2000, 2003, 2006, 2009, 2012, 2016, 2019, and 2022. The US states and hospitals included in each KID vary across years.^18^

### Main Outcome Measures

The main outcome measures included the estimated national hospital discharge rate per 100 000 children aged 0 to 18 years and the estimated national proportion of total hospital discharges, bed days, and hospital charges attributable to children with 0, 1, 2, or 3 or more CCCs. CCCs were identified with *International Classification of Diseases, 9th Revision, Clinical Modification (ICD-9-CM)* and *International Statistical Classification of Diseases and Related Health Problems, 10th Revision, Clinical Modification (ICD-10-CM)* diagnosis codes from Feudtner’s classification system, version 3.^1^ We applied the KID weighting scheme (including clustering and stratification), using HCUP instructions,^23^ to achieve national estimates with variances of the main outcomes, given the complex sampling frame of KID hospitals and unweighted discharges per hospital. Throughout the results, we consistently present weighted national estimates for all outcomes. To calculate the hospital discharge rates, population estimates for children aged 0 to 18 years, abstracted from the US Census Bureau’s Vintage system, were matched for each KID year.^24^ Hospital charges were adjusted for inflation to 2022 dollars using the medical component of the Consumer Price Index. We also adjusted the main outcome measures for changes in diagnosis coding intensity that could have occurred during the study period by using the first 5 diagnoses in each administrative claim.^16^

### Sociodemographic and Clinical Characteristics of Hospital Discharges

Sociodemographic characteristics included age category in years (<1, 1-5, 6-12, and 13-18), sex (female, male), and median household income for the patient’s zip code, stratified into quartiles. Clinical characteristics included primary diagnosis, CCC type and count, and mental health diagnosis comorbidity. The primary diagnosis category for each hospitalization was identified using the Major Diagnostic Category (MDC) categorization of principal diagnosis codes into 1 of 25 organ systems (eg, nervous, respiratory).^25^ Type of CCC included the CCC categories (eg, cardiovascular, neurologic) as well as technology dependence (eg, internal devices of the cardiac [pacemaker], gastrointestinal [gastrostomy], and neurologic [cerebrospinal fluid shunt] systems). The type of mental health diagnosis (eg, neurodevelopmental or neurocognitive disorders, anxiety disorders, or mood disorders) was identified using a grouping method^26^ of the Child and Adolescent Mental Health Disorders Classification System, which classifies diagnosis codes into child mental health conditions by the *Diagnostic and Statistical Manual of Mental Disorders*, 5th edition, psychiatric diagnosis groups.^27^ We also assessed length of stay (LOS) in days, which we categorized into groups (1-2, 3-4, 5-6, or ≥7 days), payer (public, private, or other), and hospital location and teaching status (rural, urban nonteaching, or urban teaching) for each discharge.

### Statistical Analysis

We assessed trends across the 8 KID years using weighted estimates and 95% CIs of the main outcomes to calculate percentage changes (with 95% CIs) between time points (eg, years 2000 and 2022). We assessed trends in the main outcomes with and without adjustment for diagnosis coding intensity (ie, limiting the number of individual diagnosis code slots to 5 for each hospital discharge). All analyses were completed using SAS, version 9.4 (SAS Institute Inc).

## Results

### Characteristics of the Study Population

Of the 26 342 497 weighted and estimated national hospital discharges for children aged 0 to 18 years—including complicated births—from all KIDs from 2000 to 2022 combined, 45.9% (95% CI, 45.9%-46.0%) were among females and 54.1% (95% CI, 54.0%-54.2%) among males; 55.4% (95% CI, 54.4%-55.8%) were for infants (aged 0-1 year) and 52.1% (95% CI, 51.8%-52.4%) had an LOS of less than 3 days. Of hospital discharges for infants, 14.4% (95% CI, 14.1%-14.6%) had at least 1 CCC (eTable 2 in Supplement 1). There was a decrease of 323 887 in the KID-estimated total number of US nonbirth hospital discharges from 2009 to 2012 (eTable 1 in Supplement 1). In a post hoc analysis, we excluded short-stay hospital discharges (1 night or less) from all KID years. The 2009 to 2012 findings did not change. For the KIDs from all years combined, children with 1 or more CCCs accounted for 20.1% (95% CI, 19.0%-21.1%) of the total hospital discharges. Altogether, 167 130 of the complicated births in the KID for 2022 (10.1% [95% CI, 9.8%-10.3%]) had 1 or more CCCs, representing 23.6% (95% CI, 21.5%-25.7%) of hospital discharges with 1 or more CCCs in 2022.

### Hospital Discharge Rates

#### With vs Without CCCs

**Figure 1.**
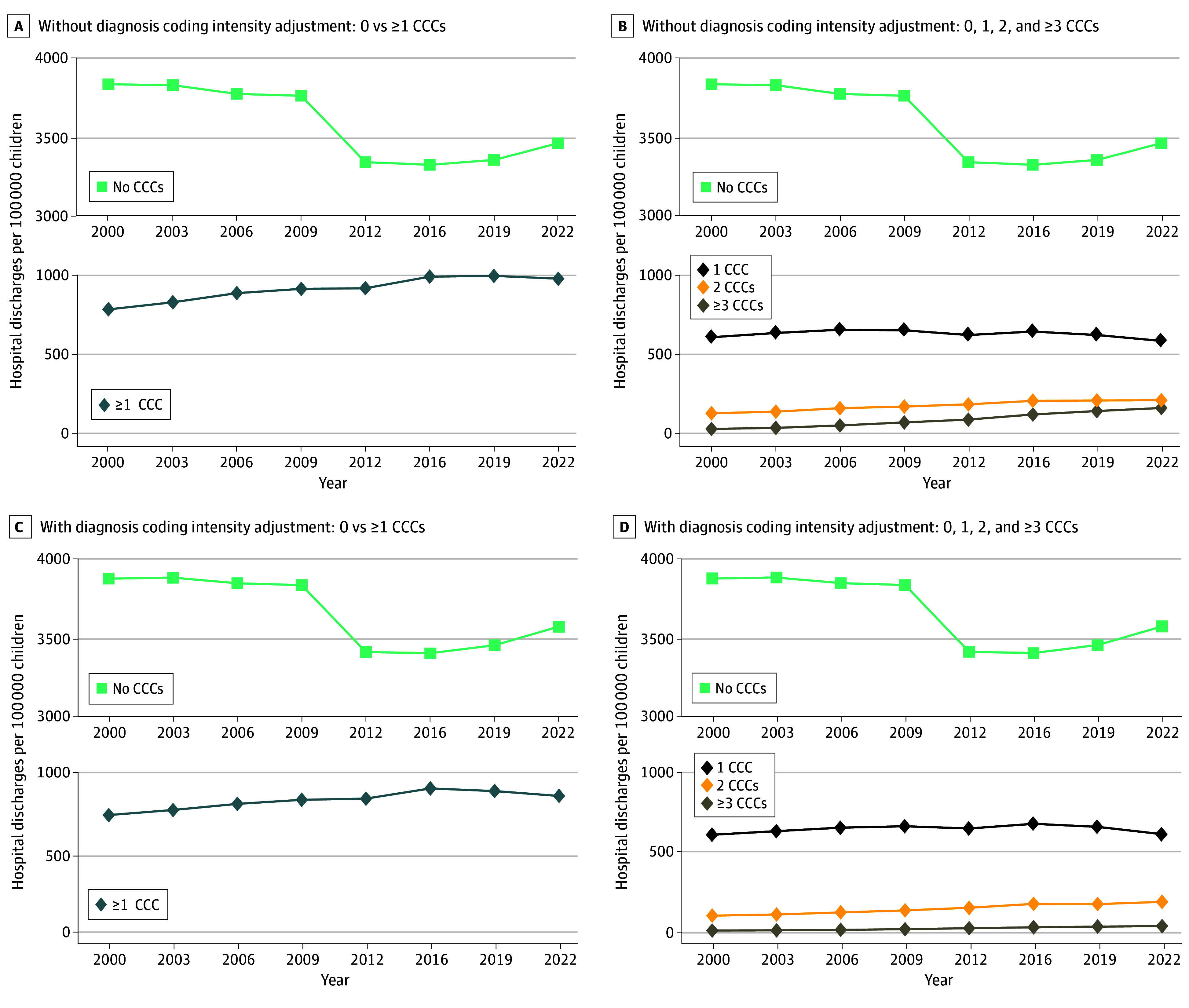
US Hospital Discharge Rate Trends for Children Aged 0 to 18 Years With and Without Complex Chronic Conditions (CCCs) Hospital discharge rates were calculated from weighted estimates from the Kids’ Inpatient Database (KID) and US Census Bureau vintage data. Years reflect all available KID data from 2000 to 2022. Diagnosis coding intensity adjustment limited the number of diagnosis codes to the first 5 in each encounter to classify hospital discharges with 0, 1, 2, or 3 or more CCCs.

From 2000 to 2022, the non-CCC hospital discharge rate per 100 000 children decreased by 9.7% (95% CI, 9.4%-10.0%), from 3831 to 3459 (Figure 1). The non-CCC hospital discharge rate decreased the most (11.1% [95% CI, 10.3%-12.0%], from 3758 to 3341) from 2009 to 2012 and then increased slightly (3.5% [95% CI, 3.5%-3.6%], from 3341 to 3459) from 2012 to 2022 (Figure 1).

In contrast, from 2000 to 2022, the hospital discharge rate per 100 000 children for children with 1 or more CCCs increased by 24.3% (95% CI, 22.7%-26.3%), from 779 to 968 (Figure 1). From 2000 to 2016, the CCC discharge rate increased by 26.1% (95% CI, 24.6%-26.3%), from 779 to 982, and then decreased from 2016 to 2022 by 1.5% (95% CI, 1.5%-1.5%), from 982 to 968 (Figure 1).

#### With Single vs Multiple CCCs

From 2000 to 2022, the percentage change in the hospital discharge rate per 100 000 children varied by number of CCCs: a 3.8% (95% CI, 0.9%-6.0%) decrease for 1 CCC (from 606 to 583), a 60.9% (95% CI, 57.7%-65.5%) increase for 2 CCCs (from 135 to 217), and a 340.0% (95% CI, 332.6%-351.1%) increase for 3 or more CCCs (from 38 to 168) (Figure 1). Adjustment for diagnosis coding intensity attenuated the hospital discharge rate of children with 3 or more CCCs the most. With adjustment, a smaller increase in the percentage change of the hospital discharge rate per 100 000 children from 2000 to 2022 occurred for children with 3 or more CCCs (176.3% [95% CI, 174.5%-179.1%] vs 340.0% [95% CI, 332.6%-351.1%]).

### Hospital Discharges, Bed Days, and Charges Attributable to Children With CCCs

#### For Children With 1 or More CCCs

**Figure 2.**
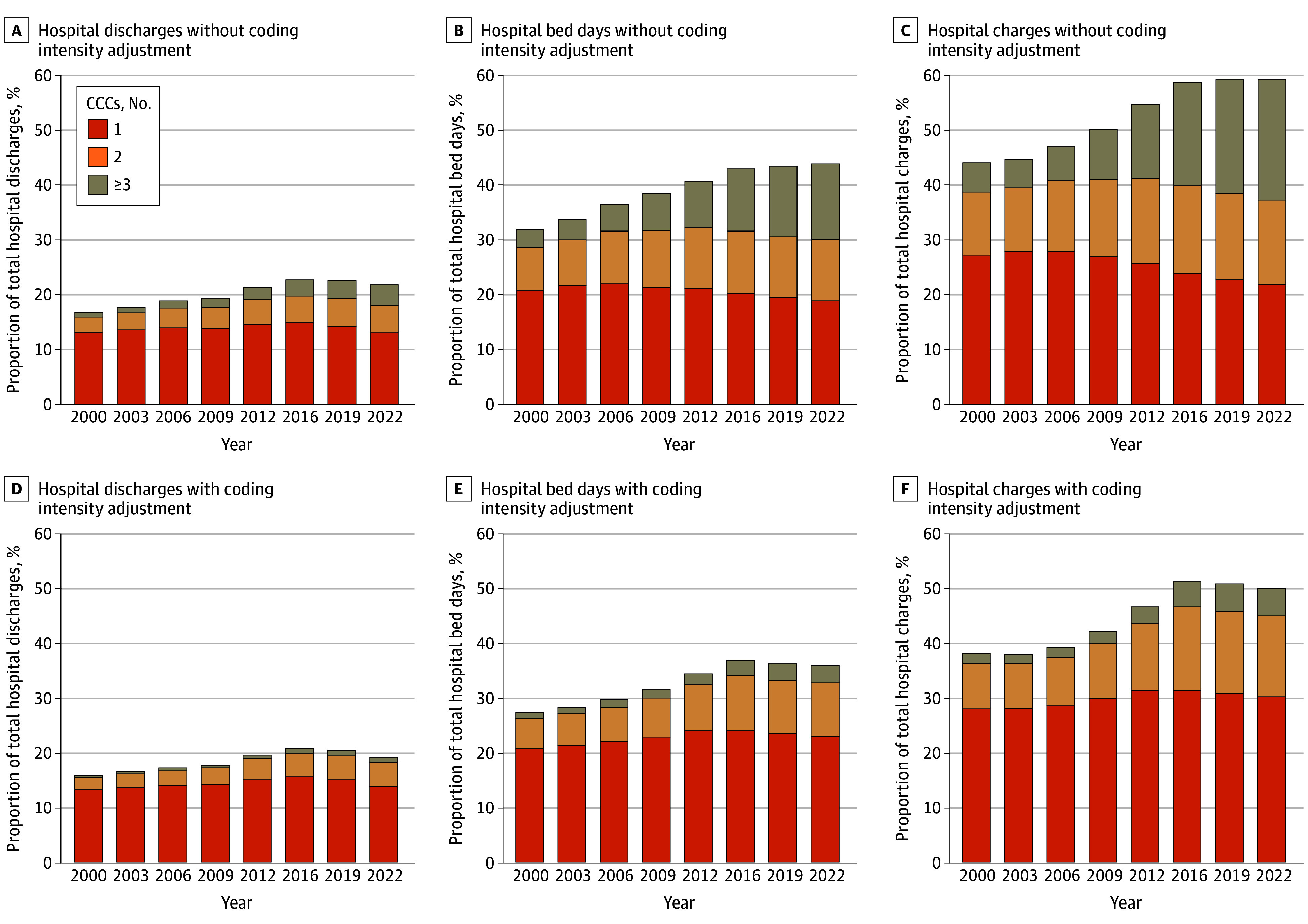
Trends in the Percentage of Hospital Discharges, Hospital Bed Days, and Hospital Charges Attributable to Children With Complex Chronic Conditions (CCCs) Percentages of total hospital resource use were calculated from weighted estimates from the Kids’ Inpatient Database (KID). Healthy-birth hospital discharges were excluded from the analysis. Years reflect all available KID data from 2000 to 2022. Hospital charges were adjusted for inflation to 2022 US dollars using the medical component of the Consumer Price Index. Diagnosis coding intensity adjustment limited the number of diagnosis codes to the first 5 in each encounter to classify hospital discharges with 0, 1, 2, or 3 or more CCCs.

From 2000 to 2022, the percentage of total hospital discharges increased by 29.4% (95% CI, 28.0%-31.3%), of bed days by 37.7% (95% CI, 37.2%-38.3%), and of charges attributable to children with 1 or more CCCs by 34.7% (95% CI, 33.8%-35.7%), with a noticeable difference in trends before and after 2016 (Figure 2). For example, the percentage of total hospital discharges attributable to children with 1 or more CCCs increased from 2000 to 2016 by 35.0% (95% CI, 33.6%-36.8%) and then decreased from 2016 to 2022 by 4.1% (95% CI, 4.1%-4.2%). The percentages of hospital bed days attributable to children with 1 or more CCCs increased by 34.7% (95% CI, 34.2%-35.3%) from 2000 to 2016 and then increased by 2.2% (95% CI, 2.2%-2.2%) from 2016 to 2022. In 2000 and 2022, children with 1 or more CCCs accounted for 16.9% (95% CI, 15.7%-17.9%) and 21.9% (95% CI, 20.7%-22.9%) of hospital discharges, 32.0% (95% CI, 30.8%-33.1%) and 44.1% (95% CI, 42.6%-45.4%) of bed days, and 44.2% (95% CI, 42.6%-45.5%) and 59.5% (95% CI, 57.8%-60.9%) of hospital charges, respectively.

#### By Number of CCCs

From 2000 to 2022, the percentage of total hospital resource use varied by number of CCCs (Figure 2). For example, the percentage of hospital bed days attributable to children with 1 CCC decreased by 9.4% (95% CI, 7.3%-11.1%). In contrast, the percentage of hospital bed days attributable to children with 2 CCCs and children with 3 or more CCCs increased by 44.5% (95% CI, 42.9%-46.4%) and 318.6% (95% CI, 312.6%-326.3%), respectively. Adjustment for diagnosis coding intensity attenuated the findings for children with 3 or more CCCs the most. With this adjustment, the percentage increase in hospital bed days attributable to children with 3 or more CCCs from 2000 to 2022 was 153.2% (95% CI, 152.1%-154.4%).

### Sociodemographic, Clinical, and Hospitalization Characteristics of Children With CCCs

**Figure 3.**
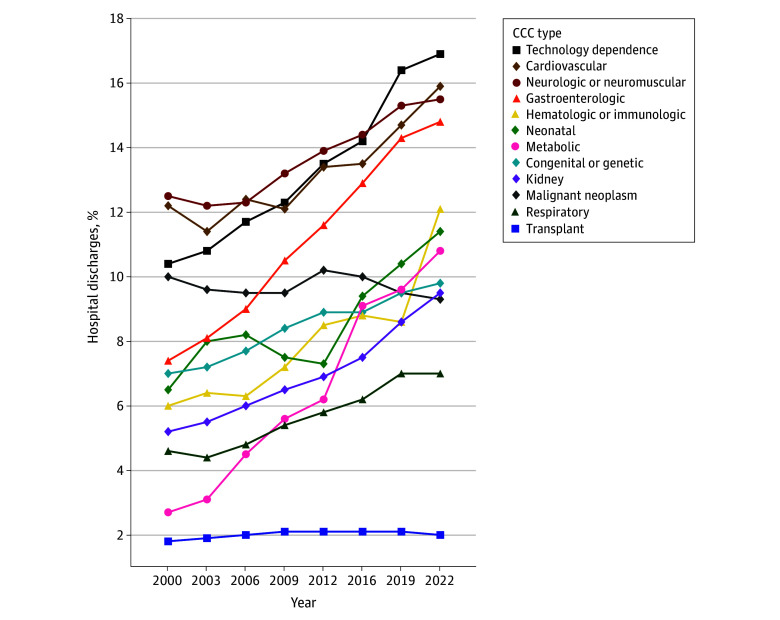
Trends in the Type of Complex Chronic Conditions (CCCs) in US Hospital Discharges of Children With CCCs CCC types are not mutually exclusive to a hospital discharge. Hospital discharges were calculated from weighted estimates from the Kids’ Inpatient Database (KID). Years reflect all available Kids’ Inpatient Database data from 2000 to 2022.

**Figure 4.**
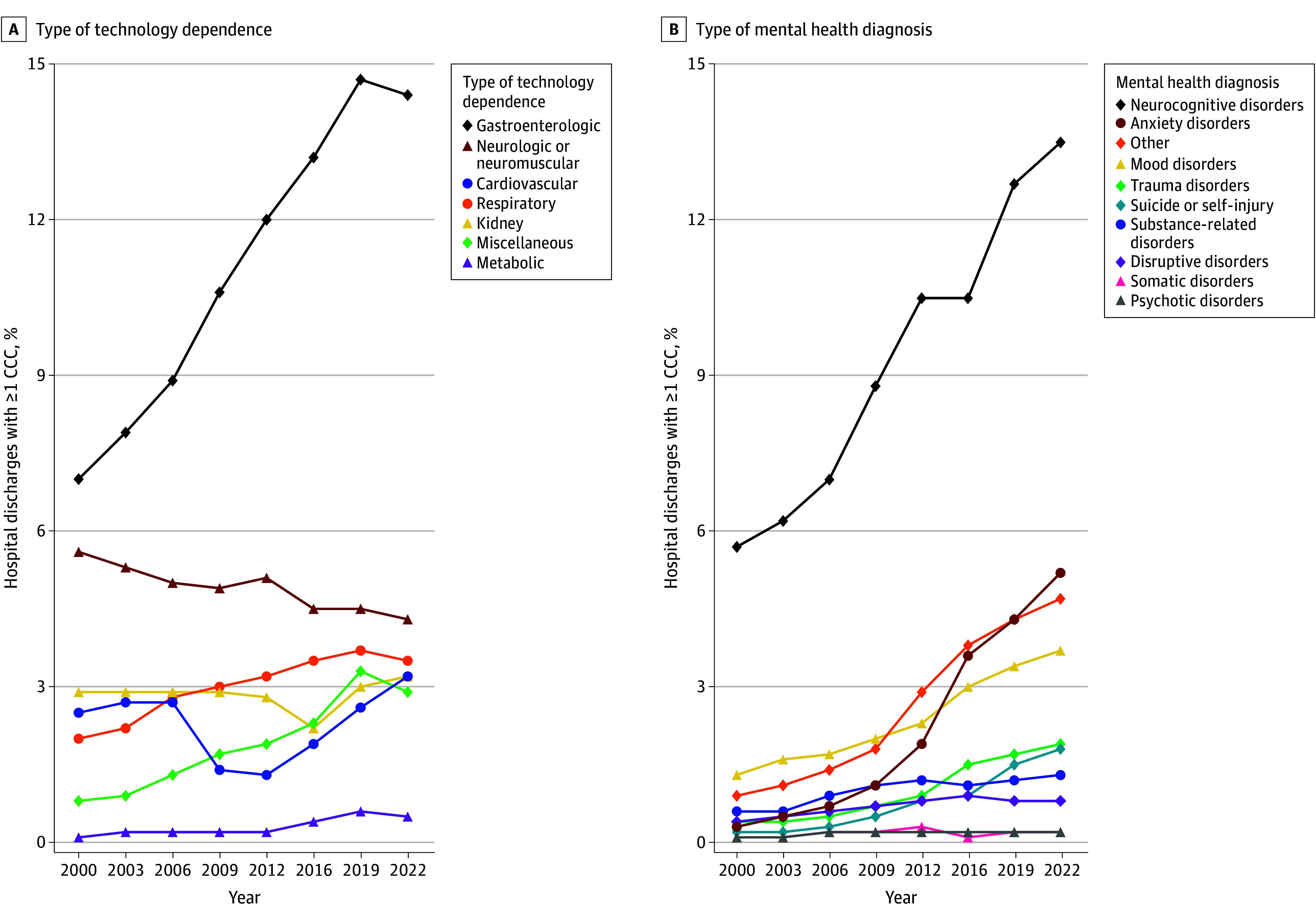
Trends in Types of Technology Dependencies and Mental Health Comorbidities in US Hospital Discharges of Children With Complex Chronic Conditions (CCCs) Type of technology dependence was distinguished with Feudtner’s CCC classification system, version 3.^1^ Type of mental health diagnosis was distinguished with the Child and Adolescent Mental Health Disorders Classification System, with categories adapted by Wolf et al.^26^ Hospital discharges were calculated from weighted estimates from the Kids’ Inpatient Database (KID). Neurodevelopmental or neurocognitive disorders include but are not limited to attention-deficit/hyperactivity disorder and intellectual disability. Years reflect all available Kids’ Inpatient Database data from 2000 to 2022. Other mental health diagnoses include autism, elimination disorders, feeding and eating disorders, sleep or wake disorders, personality disorders, dissociative disorders, and sexual or gender identity concerns.

In 2000 and 2022, respectively, 40.3% (95% CI, 38.3%-42.0%) and 40.6% (95% CI, 38.8%-42.4%) of hospital discharges for children with 1 or more CCCs were for infants (aged <1 year) (Table). The percentage of hospital discharges of children with 1 or more CCCs were higher at urban teaching hospitals (compared with rural and urban nonteaching hospitals) and increased from 78.4% (95% CI, 75.5%-81.2%) in 2000 to 94.6% (95% CI, 93.7%-95.4%) in 2022. From 2000 to 2022 for children with 1 or more CCCs, there were notable increases in the percentages of hospital discharges with public insurance as the primary payer (from 40.9% [95% CI, 38.8%-42.9%] to 52.1% [95% CI, 50.2%-54.1%]), technology dependence (from 17.9% [95% CI, 15.5%-20.3%] to 24.2% [95% CI, 20.9%-27.5%]), and mental health comorbidity (from 8.4% [95% CI, 7.3%-9.5%] to 21.9% [95% CI, 19.4%-24.5%]) (Figure 3 and Table). Examples of technologies with increases in the percentage of hospital discharges for children with CCCs from 2000 to 2022 were gastroenterologic technology (eg, gastrostomy) (from 7.0% [95% CI, 6.0%-8.0%] to 14.4% [95% CI, 12.4%-16.4%]) and respiratory technology (eg, tracheostomy) (from 2.0% [95% CI, 1.7%-2.3%] to 3.5% [95% CI, 3.0%-4.0%]) (Figure 4). Examples of types of mental health comorbidities with increases in the percentage of hospital discharges for children with CCCs were neurodevelopmental or neurocognitive disorders (from 5.7% [95% CI, 4.8%-6.5%] to 13.5% [95% CI, 11.7%-15.2%]), anxiety disorder (from 0.3% [95% CI, 0.3%-0.4%] to 5.2% [95% CI, 4.5%-5.9%]), and suicide or self-injury (from 0.4% [95% CI, 0.2%-0.3%] to 1.8% [95% CI, 1.5%-2.0%]).

**Table.** Sociodemographic, Clinical, and Hospitalization Characteristics of Hospital Discharges for Children Aged 0 to 18 Years From the Kids’ Inpatient Database, 2000 and 2022

Characteristic	Hospital discharges, No. (weighted % [95% CI])			
	CCCs		No CCCs	
	2000 (n = 563 673)	2022 (n = 707 264)	2000 (n = 2 772 940)	2022 (n = 2 528 299)
Age, y				
<1	227 172 (40.3 [38.3-42.0])	287 025 (40.6 [38.8-42.4])	1 493 429 (53.9 [52.6-54.8])	1 790 666 (70.8 [69.3-72.3])
1-5	121 890 (21.6 [20.8-22.4])	146 259 (20.7 [19.9-21.4])	488 323 (17.6 [17.1-18.1])	278 159 (11.0 [10.3-11.7])
6-12	119 245 (21.2 [20.2-22.2])	136 711 (19.3 [18.4-20.3])	388 616 (14.0 [13.5-14.5])	195 366 (7.7 [7.3-8.2])
13-18	95 366 (16.9 [16.2-17.6])	137 269 (19.4 [18.7-20.1])	402 531 (14.5 [13.9-15.2])	264 107 (10.4 [9.8-11.1])
Sex				
Female	251 755 (44.6 [44.3-44.9])	321 559 (45.5 [45.2-45.7])	1 225 016 (45.3 [45.1-45.4])	1 201 711 (47.5 [47.3-47.7])
Male	311 918 (55.4 [55.1-55.7])	385 705 (54.6 [54.3-54.8])	1 517 924 (54.8 [54.6-54.9])	1 326 588 (52.5 [52.3-52.7])
Median household income for patient’s zip code, quartile				
1 (Lowest	158 622 (27.0 [25.1-28.9])^a^	203 082 (29.0 [27.0-30.9])	805 162 (29.4 [28.0-30.8])^a^	705 841 (28.1 [26.8-29.4])
2	152 173 (25.9 [24.8-26.9])^a^	175 154 (25.0 [23.8-26.1])	707 359 (25.8 [25.0-26.7])^a^	625 623 (24.9 [24.1-25.7])
3	145 371 (24.7 [23.7-25.7])^a^	171 353 (24.4 [23.5-25.4])	649 430 (23.7 [22.9-24.5])^a^	623 202 (24.8 [24.1-25.5])
4 (Highest)	132 012 (22.4 [20.5-24.4])^a^	151 455 (21.6 [19.5-23.7])	578 142 (21.1 [19.8-22.4])^a^	557 784 (22.2 [20.8-23.6])
Mental health comorbidity	47 259 (8.4 [7.8-9.1])	144 836 (20.5 [21.2-22.7])	239 726 (8.6 [8.0-9.6])	340 172 (13.5 [12.7-14.3])
4 Most common primary diagnoses by MDC^b^				
Newborn conditions during perinatal period	134 541 (23.9 [21.8-26.0])	198 973 (28.1 [26.0-30.3])	1 073 951 (38.8 [37.3-40.1])	1 602 978 (63.4 [61.5-65.3])
Respiratory system	56 488 (10.0 [9.6-10.5])	88 838 (12.6 [12.1-13.0])	463 915 (16.7 [16.2-17.3])	311 034 (12.3 [11.5-13.1])
Nervous system	58 850 (10.4 [9.9-11.0])	67 263 (9.5 [9.0-10.0])	106 379 (3.8 [3.6-4.0])	56 837 (2.3 [2.1-2.4])
Digestive system	41 804 (7.4 [7.1-7.7])	48 479 (6.9 [6.6-7.2])	258 999 (9.3 [9.1-9.6])	86 678 (3.4 [3.2-3.7])
Length of stay, d				
1-2	203 600 (36.1 [35.2-37.1])	259 056 (36.6 [35.6-37.6])	1 539 260 (55.5 [54.8-56.2])	1 540 536 (60.9 [60.2-61.7])
3-4	129 521 (23.0 [22.6-23.4])	153 421 (21.7 [21.3-22.0])	705 816 (25.5 [25.1-25.8])	545 745 (21.6 [21.2-22.0])
5-6	64 004 (11.4 [11.0-11.7])	74 304 (10.5 [10.2-10.8])	200 388 (7.2 [7.0-7.4])	157 237 (6.2 [6.0-6.5])
≥7	166 547 (29.5 [28.7-30.4])	220 482 (31.2 [30.5-31.8])	327 434 (11.8 [11.4-12.2])	284 780 (11.3 [10.9-11.6])
Hospital location				
Rural	31 304 (5.6 [4.4-6.8])	14 127 (2.0 [1.4-2.5])	408 769 (14.8 [13.8-15.8])	160 218 (6.3 [5.8-6.9])
Urban				
Nonteaching	89 871 (16.0 [13.6-18.4])	24 334 (3.4 [2.9-4.0])	926 173 (33.5 [31.6-35.4])	261 301 (10.3 [9.5-11.2])
Teaching	439 294 (78.4 [75.5-81.2])	668 804 (94.6 [93.7-95.4])	1 429 793 (51.7 [49.6-53.8])	2 106 778 (83.3 [82.2-84.4])
Payer				
Public	230 288 (40.9 [38.8-42.9])	368 709 (52.1 [50.2-54.1])	1 044 558 (37.7 [36.4-38.9])	1 247 177 (49.3 [48.2-50.4])
Private	294 457 (52.2 [50.1-54.4])	279 739 (39.6 [38.2-40.9])	1 491 396 (53.8 [52.3-55.2])	1 084 211 (42.9 [41.8-44.0])
Other^c^	38 928 (6.9 [5.8-7.4])	58 816 (8.3 [6.5-9.8])	236 944 (8.5 [7.5-8.8])	196 909 (7.8 [7.2-8.2])

Abbreviations: CCC, complex chronic condition; MDC, major diagnostic category.

^a^ Data presented are from 2003, as 2000 data were incomparable due to changes in Kids’ Inpatient Database methods.

^b^ Chosen from the most common MDCs in 2022 for CCC hospital discharges.

^c^ Included self-pay and no charge to the patient for the hospital discharge.

## Discussion

The findings from the current study suggest that the US hospital discharge rate and percentage of hospital discharges, bed days, and charges attributable to children with 1 or more CCCs increased from 2000 to 2022 and that these trends were driven by the increase in hospital discharges and hospital resource use for children with multiple CCCs. Although adjustment for diagnosis coding intensity attenuated the findings for hospitalized children with multiple CCCs the most, the trends of increasing hospital resource use in this population persisted and remained substantial. Over time, the profile of demographic and clinical characteristics of hospitalized children with CCCs changed, with a greater presence of Medicaid use, gastroenterologic technology dependence, and neurodevelopmental disorders in particular.

The findings from the current study both compare and contrast with prior literature. The CCC hospital discharge rates per 100 000 children ages 0 to 18 years reported in the current study from 2000 to 2006 are congruent with the rates reported by Burns et al^11^ from the AHRQ’s Nationwide Inpatient Sample. Burns et al^11^ restricted their study sample to children who were 8 days to 4 years of age. We observed a higher percentage of hospital discharges, bed days, and charges for children with CCCs in the KID from 2000 to 2006 than reported by Simon et al^12^ in the same KID years. Simon et al^12^ included healthy birth hospitalizations, which were excluded in the present study. We observed a lower percentage of hospital resource use attributable to children with CCCs than reported by Leyenaar et al^15^ in the KID from years 2009 to 2022. Leyenaar et al^15^ excluded all birth hospital discharges in their primary analysis. We retained complicated births, because some could have been for newborns with CCCs (eg, complex chronic congenital anomaly), who use substantial hospital and neonatal intensive care resources. In a post hoc analysis, 10.1% (95% CI, 9.85%-10.3%) of the complicated births in the KID for 2022 had 1 or more CCCs, representing 23.6% (95% CI, 21.5%-25.7%) of hospital discharges with 1 or more CCCs in 2022. We applied the most recent version of Feudtner’s diagnosis code classification system (version 3, released in 2024, which reconceptualized the system’s use of technology dependence codes) to distinguish hospital discharges with a CCC, and this could have influenced identified trends.^1^

The current study revealed an unexpected inflection point from 2009 to 2012 in trends in hospital discharge rates, especially for children without a CCC. The reasons for the unexpected 323 887-count decrease in the KID-estimated total number of US nonbirth hospital discharges from 2009 to 2012 are speculative. National policy changes in classification of short-stay hospitalizations from inpatient to observation status, which emerged in the beginning of the 2010 decade, may have contributed.^20,21,28,29^ The KID does not include inpatient stays for observation. In a post hoc analysis, we excluded short-stay hospital discharges (ie, 1-night LOS or less) from all KID years, and the 2009 to 2012 findings did not change. From 2009 to 2012, the AHRQ improved its sampling strategy for a related but different inpatient database, the National Inpatient Sample, which resulted in a 1-time disruption to historical trends, including a decline in discharge counts beginning in 2012.^30^ No corresponding revision to the KID sampling strategy occurred (email correspondence, AHRQ HCUP support team, August 15, 2025).

Further investigation is needed to explain the tempered trends, starting with the 2016 KID, for hospital resource use in children with CCCs. In the KID from 2016 to 2022, the hospital discharge rate for children without a CCC increased and the percentage change of hospital resources attributable to children with CCCs dwindled. The 2016 KID was the first to contain *ICD-10-CM* diagnosis codes, following the transition from *ICD-9-CM* in October 2015.^31^ Although we applied both *ICD-9-CM* and *ICD-10-CM* versions of Feudtner’s system^1^ to distinguish hospital discharges with a CCC, the transition still could have influenced the trends. The COVID-19 pandemic was still active with variant surges in 2022. Some families of children with CCCs may have continued engagement in isolation and other preventive measures, which may have mitigated hospital resource use more than for children without CCCs.^32^ Changes to the KID sampling frame could also have contributed. Compared with prior years, the 2016-2022 KID had the fewest number of states added or removed.^33^

Improved survival merits attention as a unifying factor that may have contributed to several of the findings of the current study, including increased hospital discharges for children with multiple CCCs as well as a rising prevalence of neurodevelopmental disorders and gastrointestinal technology dependence in hospitalized children with CCCs. For example, survival for complex congenital heart disease, including single-ventricle physiology, has increased as much as 33% over past decades.^34^ In survivors of complex congenital heart disease, neurodevelopmental disorders (eg, intellectual disability) and undernutrition are prevalent, consequential morbidities that can necessitate gastrostomy.^35,36^ These survivors are also at risk for complex, chronic pulmonary problems (eg, airway compression, disruption of the pulmonary vascular system).^37^ Families of infants with complex congenital heart disease report insufficient support with caregiving at home.^38^ All of these attributes—underpinned by increasing survival—may help explain the high prevalence of multiple CCCs and increasing hospital resource use reported for children with congenital heart disease.^39^ Assessment of survival and impact on hospital resource use with other CCCs is warranted.

The findings from the current study highlight a 20-year progression of increasing hospital discharge rates and hospital resource use attributable to children with CCCs, especially multiple CCCs. The evolving shift in the inpatient pediatric case mix toward more multiorgan system involvement, technology dependence, mental health comorbidities, and Medicaid use has important implications. First, regarding medical education, recent Accreditation Council for Graduate Medical Education revisions for pediatric residency programs include decreased inpatient exposures for patient care and learning.^40^ The effect of these revisions on the ability of pediatric residents to achieve competency in the care for hospitalized children with CCCs will be important to assess. Currently, pediatric residents report increasing unpreparedness to independently practice in the inpatient setting.^41^ Second, evaluations of the structure, personnel, and process of inpatient clinical teams are needed to distinguish best practices for delivering high-quality care to hospitalized children with CCCs. Examples of these team evaluations include general hospitalist vs specialty-based teams, comanagement and consultative models, teams exclusively admitting children with CCCs, and integration with outpatient and home care programs.

Third, in light of increased Medicaid use in hospitalized children with CCCs, inpatient clinical teams and hospitals must assess their staffing and resources available to help with the medical, social, and family challenges that children with CCCs experience, especially when transitioning from hospital to home. Caregivers of children with CCCs report substantial employment challenges, financial hardships, mental health challenges, and insufficient home care and respite services—all of which hospitalization can exacerbate.^42,43,44,45^ Increasing evidence supports that health systems—including inpatient—must simultaneously address medical and social needs to optimize health and well-being for children with CCCs.^46,47^ In addition, health systems and future research should assess the impact of increased Medicaid use in children with CCCs on the financial solvency of pediatric inpatient services. Underpayment by Medicaid not covering hospitalization costs,^48,49^ decreased federal spending on Medicaid from recent legislation,^50^ and increased closures of pediatric inpatient units in general hospitals, including those in rural areas,^48,51,52,53^ may challenge and strain hospitals—especially children’s and teaching hospitals—to care for children with CCCs.

### Limitations

The current study has several limitations. The KID was available as a standalone dataset for 8 of the 23 years in the study period, with repeated changes in contributing states and hospitals. This limits the interpretation of the trends and the ability to assess the impact of important events (eg, the COVID-19 pandemic). We could not determine the number of children responsible for the hospital resource use, because the KID does not permit tracking of individual patients across multiple hospital discharges. US Census data do not provide population estimates of children with CCCs, so the hospital discharge rates were calculated from all children. The KID also does not contain hospital discharges classified with an observation status. Inclusion of such discharges could affect the findings. The current study was not intended to predict future hospital resource use for children with CCCs. Therefore, the historical trends were presented in an unadjusted, population-based, stratified form rather than a case-mix adjusted form for type and count of CCCs as well as for sociodemographic characteristics. The findings on hospital charges do not necessarily reflect costs or reimbursements for hospital care. Further investigation is needed to assess the generalizability of the findings of children with CCCs to children with medical complexity (CMC). When coupled with information on functional status, health care needs (eg, chronic use of medication and durable medical equipment), and health resource use beyond the hospital, it is possible that some of the hospital discharges for children with multiple CCCs and/or technology dependence may generalize best to CMC.^2,54^

## Conclusions

In this national repeated cross-sectional study, hospital discharge rates and the percentage of hospital discharges, bed days, and charges for children with CCCs increased substantially from 2000 to 2022. The findings suggest children with multiple CCCs were the major driver of the observed trends in increased hospital resource use. Technology dependence (especially gastrointestinal technology) and comorbid mental health conditions (especially neurodevelopmental disorders) became more prevalent among hospitalized children with CCCs. These findings suggest that the population of hospitalized children with CCCs became more medically complex over time. It is critical that health systems are sufficiently equipped with the resources, staff, and payments to sustainably meet these children’s increasing needs for inpatient care.

## Notes

Supplement 1. **eTable 1.** Sample Setting and Study Population of Hospital Discharges by Year of the Kids’ Inpatient Database **eTable 2.** National Estimates of Sociodemographic and Clinical Characteristics of Hospital Discharges for Children Aged 0 to 18 Years in the Kids’ Inpatient Database, 2000, 2003, 2006, 2009, 2012, 2016, 2019, and 2022

Supplement 2.
Data Sharing Statement

## References

[1] Feinstein JA, Hall M, Davidson A, Feudtner C. Pediatric complex chronic condition system version 3. JAMA Netw Open. 2024;7(7):e2420579. doi:10.1001/jamanetworkopen.2024.20579 39008301 PMC11250371

[2] Berry JG, Hall M, Neff J, . Children with medical complexity and Medicaid: spending and cost savings. Health Aff (Millwood). 2014;33(12):2199-2206. doi:10.1377/hlthaff.2014.0828 25489039 PMC5164920

[3] Leyenaar JK, Arakelyan M, Schaefer AP, . Neurodevelopmental and mental health conditions in children with medical complexity. Pediatrics. 2024;154(3):e2024065650. doi:10.1542/peds.2024-065650 39099441 PMC11350095

[4] Blaine K, Rogers J, OʼNeill MR, . Clinician perceptions of the importance of hospital discharge components for children. J Healthc Qual. 2018;40(2):79-88. doi:10.1097/JHQ.0000000000000084 29329135

[5] Berry JG, Glotzbecker M, Rodean J, Leahy I, Hall M, Ferrari L. Comorbidities and complications of spinal fusion for scoliosis. Pediatrics. 2017;139(3):e20162574. doi:10.1542/peds.2016-2574 28153850 PMC5330399

[6] Gold JM, Hall M, Shah SS, . Long length of hospital stay in children with medical complexity. J Hosp Med. 2016;11(11):750-756. doi:10.1002/jhm.2633 27378587

[7] Berry JG, Hall M, Dumas H, . Pediatric hospital discharges to home health and postacute facility care: a national study. JAMA Pediatr. 2016;170(4):326-333. doi:10.1001/jamapediatrics.2015.4836 26902773

[8] Berry JG, Toomey SL, Zaslavsky AM, . Pediatric readmission prevalence and variability across hospitals. JAMA. 2013;309(4):372-380. doi:10.1001/jama.2012.188351 23340639 PMC3640861

[9] Berry JG, Hall DE, Kuo DZ, . Hospital utilization and characteristics of patients experiencing recurrent readmissions within children’s hospitals. JAMA. 2011;305(7):682-690. doi:10.1001/jama.2011.122 21325184 PMC3118568

[10] Bjur KA, Wi CI, Ryu E, Crow SS, King KS, Juhn YJ. Epidemiology of children with multiple complex chronic conditions in a mixed urban-rural US community. Hosp Pediatr. 2019;9(4):281-290. doi:10.1542/hpeds.2018-0091 30923070 PMC6434974

[11] Burns KH, Casey PH, Lyle RE, Bird TM, Fussell JJ, Robbins JM. Increasing prevalence of medically complex children in US hospitals. Pediatrics. 2010;126(4):638-646. doi:10.1542/peds.2009-1658 20855383

[12] Simon TD, Berry J, Feudtner C, . Children with complex chronic conditions in inpatient hospital settings in the United States. Pediatrics. 2010;126(4):647-655. doi:10.1542/peds.2009-3266 20855394 PMC2962571

[13] Zima BT, Rodean J, Hall M, Bardach NS, Coker TR, Berry JG. Psychiatric disorders and trends in resource use in pediatric hospitals. Pediatrics. 2016;138(5):e20160909. doi:10.1542/peds.2016-0909 27940773 PMC5079078

[14] Hall M, Berry JG, Hall M, . Changes in hospitalization populations by level of complexity at children’s hospitals. J Hosp Med. 2024;19(5):399-402. doi:10.1002/jhm.13292 38340352

[15] Leyenaar JK, Freyleue S, Arakelyan M, Schaefer AP. Hospitalizations by children with medical complexity from 2009 to 2022. Pediatrics. 2025;156(2):e2025071774. doi:10.1542/peds.2025-071774 40675610

[16] Tsugawa Y, Figueroa JF, Papanicolas I, Orav EJ, Jha AK. Assessment of strategies for managing expansion of diagnosis coding using risk-adjustment methods for Medicare data. JAMA Intern Med. 2019;179(9):1287-1290. doi:10.1001/jamainternmed.2019.1005 31242282 PMC6596333

[17] Crespin D, Dworsky M, Levin J, Ruder T, Whaley CM. Upcoding linked to up to two-thirds of growth in highest-intensity hospital discharges in 5 states, 2011-19. Health Aff (Millwood). 2024;43(12):1619-1627. doi:10.1377/hlthaff.2024.00596 39626153

[18] Healthcare Cost and Utilization Project (HCUP). Introduction to the HCUP Kids’ Inpatient Database (KID) 2022. December 2024. Accessed August 13, 2025. https://hcup-us.ahrq.gov/db/nation/kid/KIDIntroduction2022.pdf

[19] Healthcare Cost and Utilization Project (HCUP). Kids’ Inpatient Database (KID) database documentation. Agency for Healthcare Research and Quality. December 2024. Accessed January 20, 2025. http://www.hcup-us.ahrq.gov/db/nation/kid/kiddbdocumentation.jsp

[20] Locke C, Sheehy AM, Deutschendorf A, Mackowiak S, Flansbaum BE, Petty B. Changes to inpatient versus outpatient hospitalization: Medicare’s 2-midnight rule. J Hosp Med. 2015;10(3):194-201. doi:10.1002/jhm.2312 25557865

[21] Macy ML, Hall M, Shah SS, . Pediatric observation status: are we overlooking a growing population in children’s hospitals? J Hosp Med. 2012;7(7):530-536. doi:10.1002/jhm.1923 22371384

[22] von Elm E, Altman DG, Egger M, Pocock SJ, Gøtzsche PC, Vandenbroucke JP; STROBE Initiative. The Strengthening the Reporting of Observational Studies in Epidemiology (STROBE) statement: guidelines for reporting observational studies. PLoS Med. 2007;4(10):e296. doi:10.1371/journal.pmed.0040296 17941714 PMC2020495

[23] Chu B, Houchens R, Elixhauser A, Ross D. Using the KIDS’ Inpatient Database (KID) to estimate trends. Healthcare Cost and Utilization Project Methods Series report #2007-02. January 10, 2007. Agency for Healthcare Research and Quality. Accessed October 29, 2025. http://www.hcup-us.ahrq.gov/reports/methods.jsp

[24] US Census Bureau. Explore census data. US Department of Commerce. Accessed August 13, 2025. https://data.census.gov/

[25] Centers for Medicare & Medicaid Services. Design and development of the Diagnosis Related Group (DRG). Accessed February 11, 2023. https://www.cms.gov/icd10m/version37-fullcode-cms/fullcode_cms/Design_and_development_of_the_Diagnosis_Related_Group_(DRGs).pdf

[26] Wolf RM, Hall M, Williams DJ, . Disparities in pharmacologic restraint for children hospitalized in mental health crisis. Pediatrics. 2024;153(1):e2023061353. doi:10.1542/peds.2023-061353 38073320 PMC10764008

[27] Zima BT, Gay JC, Rodean J, . Classification system for International Classification of Diseases, Ninth Revision, Clinical Modification and Tenth Revision Pediatric Mental Health Disorders. JAMA Pediatr. 2020;174(6):620-622. doi:10.1001/jamapediatrics.2020.0037 32202603 PMC7091372

[28] Tian Y, Macy ML, Hockenberry JM, . Trends in preventable hospitalization rates for children with or without observation stay data. JAMA Netw Open. 2025;8(3):e251533. doi:10.1001/jamanetworkopen.2025.1533 40126481 PMC11933988

[29] Feng Z, Wright B, Mor V. Sharp rise in Medicare enrollees being held in hospitals for observation raises concerns about causes and consequences. Health Aff (Millwood). 2012;31(6):1251-1259. doi:10.1377/hlthaff.2012.0129 22665837 PMC3773225

[30] Healthcare Cost & Utilization Project. Introduction to the HCUP National Inpatient Sample (NIS) 2012. Agency for Healthcare Research and Quality. June 2014. Updated November 2015. Accessed August 13, 2025. https://hcup-us.ahrq.gov/db/nation/nis/NIS_Introduction_2012.jsp

[31] Hirsch JA, Nicola G, McGinty G, . *ICD-10*: history and context. AJNR Am J Neuroradiol. 2016;37(4):596-599. doi:10.3174/ajnr.A4696 26822730 PMC7960170

[32] Asan O, Elkourdi F, Super I, Rezaeian O, Percy S, Clouser K. Children with medical complexity care journey during COVID-19 from providers perspective: a qualitative study. BMC Health Serv Res. 2025;25(1):740. doi:10.1186/s12913-025-12857-9 40399952 PMC12096769

[33] Agency for Healthcare Research and Quality. National Healthcare Quality & Disparities Report Chartbooks. 2023. Accessed August 13, 2025. https://www.ahrq.gov/research/findings/nhqrdr/chartbooks/index.html

[34] Stephens SB, Morris SA, Benjamin RH, . Longitudinal trends in pediatric survival by congenital heart defect in Texas, 1999 to 2017. JACC Adv. 2025;4(6 pt 1):101812. doi:10.1016/j.jacadv.2025.101812 40393281 PMC12149392

[35] Miller TA, Sharma B, Gongwer R, ; Pediatric Heart Network Investigators. Neurodevelopmental outcomes in early adolescence: the Pediatric Heart Network single ventricle reconstruction trial. Circulation. Published online July 17, 2025. doi:10.1161/CIRCULATIONAHA.125.074523 40671650 PMC12321229

[36] Mahdi EM, Tran NN, Ourshalimian S, . Factors impacting long-term gastrostomy tube dependence in infants with congenital heart disease. J Surg Res. 2022;270:455-462. doi:10.1016/j.jss.2021.09.023 34800791

[37] Healy F, Hanna BD, Zinman R. Pulmonary complications of congenital heart disease. Paediatr Respir Rev. 2012;13(1):10-15. doi:10.1016/j.prrv.2011.01.007 22208788

[38] Imperial-Perez F, Heilemann MV. Having to be the one: mothers providing home care to infants with complex cardiac needs. Am J Crit Care. 2019;28(5):354-360. doi:10.4037/ajcc2019887 31474605

[39] Edelson JB, Rossano JW, Griffis H, . Resource use and outcomes of pediatric congenital heart disease admissions: 2003 to 2016. J Am Heart Assoc. 2021;10(4):e018286. doi:10.1161/JAHA.120.018286 33554612 PMC7955343

[40] Accreditation Council for Graduate Medical Education. ACGME program requirements for graduate medical education in pediatrics. February 4, 2024. Accessed August 20, 2025. https://www.acgme.org/globalassets/pfassets/programrequirements/2025-reformatted-requirements/320_pediatrics_2025_reformatted.pdf

[41] Gottschlich EA, Frintner MP, Kist TW, Haftel HM. Pediatric residents’ preparedness and training satisfaction: 2015 to 2022. Pediatrics. 2024;153(2):e2023063764. doi:10.1542/peds.2023-063764 38258395

[42] Kuo DZ, Cohen E, Agrawal R, Berry JG, Casey PH. A national profile of caregiver challenges among more medically complex children with special health care needs. Arch Pediatr Adolesc Med. 2011;165(11):1020-1026. doi:10.1001/archpediatrics.2011.172 22065182 PMC3923457

[43] Thomson J, Shah SS, Simmons JM, . Financial and social hardships in families of children with medical complexity. J Pediatr. 2016;172:187-193.e1. doi:10.1016/j.jpeds.2016.01.049 26897040 PMC4846519

[44] Wright SM, Zaniletti I, Goodwin EJ, . Income and household material hardship in children with medical complexity. Hosp Pediatr. 2024;14(4):e195-e200. doi:10.1542/hpeds.2023-007563 38487829

[45] Kusma JD, Davis MM, Foster C. Characteristics of Medicaid policies for children with medical complexity by state: a qualitative study. JAMA Netw Open. 2022;5(10):e2239270. doi:10.1001/jamanetworkopen.2022.39270 36315145 PMC9623434

[46] Berry JG, Harris D, Coller RJ, . The interwoven nature of medical and social complexity in US children. JAMA Pediatr. 2020;174(9):891-893. doi:10.1001/jamapediatrics.2020.0280 32338723 PMC7186912

[47] Pankewicz A, Davis RK, Kim J, . Children with special needs: social determinants of health and care coordination. Clin Pediatr (Phila). 2020;59(13):1161-1168. doi:10.1177/0009922820941206 32672059

[48] Cushing AM, Bucholz EM, Chien AT, Rauch DA, Michelson KA. Availability of pediatric inpatient services in the United States. Pediatrics. 2021;148(1):e2020041723. doi:10.1542/peds.2020-041723 34127553 PMC8642812

[49] Colvin JD, Hall M, Berry JG, . Financial loss for inpatient care of Medicaid-insured children. JAMA Pediatr. 2016;170(11):1055-1062. doi:10.1001/jamapediatrics.2016.1639 27618284

[50] Gaffney A, Himmelstein DU, Woolhandler S. Projected effects of proposed cuts in federal Medicaid expenditures on Medicaid enrollment, uninsurance, health care, and health. Ann Intern Med. 2025;178(9):1334-1342. doi:10.7326/ANNALS-25-00716 40523288

[51] Leyenaar JK, Ralston SL, Shieh MS, Pekow PS, Mangione-Smith R, Lindenauer PK. Epidemiology of pediatric hospitalizations at general hospitals and freestanding children’s hospitals in the United States. J Hosp Med. 2016;11(11):743-749. doi:10.1002/jhm.2624 27373782 PMC5467435

[52] Shah S, Kuo AA, Brumberg HL. First aid for Medicaid: losses in children’s health insurance. Pediatr Res. 2021;89(1):8-11. doi:10.1038/s41390-020-01219-2 33219326 PMC7678570

[53] Arakelyan M, Freyleue SD, Schaefer AP, . Rural-urban disparities in health care delivery for children with medical complexity and moderating effects of payer, disability, and community poverty. J Rural Health. 2024;40(2):326-337. doi:10.1111/jrh.12827 38379187 PMC10954394

[54] Berry JG, Hall M, Cohen E, O’Neill M, Feudtner C. Ways to identify children with medical complexity and the importance of why. J Pediatr. 2015;167(2):229-237. doi:10.1016/j.jpeds.2015.04.068 26028285 PMC5164919

