# Supplementary material for: Trends in Hospital Resource Use for Children With Complex Chronic Conditions
Source: JAMA Netw Open. 2025 Dec 2;8(12):e2544686. doi: 10.1001/jamanetworkopen.2025.44686 (PMC12673417; doi:10.1001/jamanetworkopen.2025.44686)
Supplement: Supplement 1. — eTable 1. Sample Setting and Study Population of Hospital Discharges by Year of the Kids’ Inpatient Database eTable 2. National Estimates of Sociodemographic and Clinical Characteristics of Hospital Discharges for Children Aged 0 to 18 Years in the Kids’ Inpatient Database, 2000, 2003, 2006, 2009, 2012, 2016, 2019, and 2022 [file jamanetwopen-e2544686-s001.pdf]

Supplementary Online Content

Bayer ND, Hall M, Osipovich M, et al. Trends in hospital resource use for children with complex chronic conditions. *JAMA Netw Open*. 2025;8(12):e2544686. doi:10.1001/jamanetworkopen.2025.44686

eTable 1. Sample Setting and Study Population of Hospital Discharges by Year of the Kids' Inpatient Database
eTable 2. National Estimates of Sociodemographic and Clinical Characteristics of Hospital Discharges for Children Aged 0 to 18 Years in the Kids' Inpatient Database, 2000, 2003, 2006, 2009, 2012, 2016, 2019, and 2022

This supplementary material has been provided by the authors to give readers additional information about their work.

© 2025 Bayer ND et al. *JAMA Network Open*.

eTable 1. Sample Setting and Study Population of Hospital Discharges by Year of the Kids' Inpatient Database

Characteristic	2000	2003	2006	2009	2012	2016	2019	2022
U.S. states	27	36	38	44	44	47	49	48
Hospitals	2,784	3,438	3,739	4,121	4,179	4,200	3,998	3,811
**Hospital Discharges for All Births and Non-Births for Ages 0-to-21 Years (All Discharges)**								
Unweighted	2,516,833	2,984,129	3,131,324	3,407,146	3,195,782	3,117,413	3,089,283	3,009,812
Weighted	7,291,039	7,409,312	7,558,821	7,370,203	6,675,222	6,266,285	5,902,538	5,660,863
**Weighted Hospital Discharges Exclusions[1]**								
(#1) Ages 18-to-21 years	6,358,295 (87.2%)	6,508,763 (87.8%)	6,614,939 (87.5%)	6,426,989 (87.2%)	5,859,250 (87.8%)	5,604,984 (89.4%)	5,299,557 (89.8%)	5,140,396 (90.8%)
(#2) Pregnancy, Childbirth, and the Puerperium[2]	6,161,117 (84.5%)	6,336,706 (85.5%)	6,439,080 (85.2%)	6,270,520 (85.1%)	5,753,838 (86.2%)	5,539,421 (88.4%)	5,249,667 (88.9%)	5,097,577 (90.0%)
(#3) Uncomplicated Births[3]	3,336,572 (45.8%)	3,397,041 (45.8%)	3,429,637 (45.4%)	3,457,303 (46.9%)	3,133,417 (46.9%)	3,173,956 (50.7%)	3,179,010 (53.9%)	3,235,561 (57.2%)
**Weighted Hospital Discharges, Bed Days, and Charges of the Retained Study Population [n (%)]**								
**Discharges**	**3,336,572**	**3,397,041**	**3,429,637**	**3,457,303**	**3,133,417**	**3,173,956**	**3,179,010**	**3,235,561**
No CCC	2,772,940 (83.1)	2,795,596 (82.3)	2,780,818 (81.1)	2,786,038 (80.6)	2,462,954 (78.6)	2,450,219 (77.2)	2,456,804 (77.3)	2,528,299 (78.1)
1 CCC	438,518 (13.1)	461,646 (13.6)	480,869 (14.0)	480,820 (13.9)	458,049 (14.6)	472,651 (14.9)	455,095 (14.3)	426,009 (13.2)
2 CCCs	97,453 (2.9)	106,389 (3.1)	122,933 (3.6)	132,248 (3.8)	140,887 (4.5)	156,625 (4.9)	157,699 (5.0)	158,353 (4.9)
≥3 CCCs	27,661 (0.8)	33,410 (1.0)	45,017 (1.3)	58,197 (1.7)	71,526 (2.3)	94,460 (3.0)	109,412 (3.4)	122,901 (3.8)
**Bed Days (millions)**	**16.3**	**16.9**	**17.8**	**18.0**	**16.9**	**17.6**	**17.6**	**17.8**
No CCC	11.1 (68.0)	11.1 (66.2)	11.3 (63.4)	11.0 (61.5)	10.0 (59.1)	10.0 (56.9)	9.9 (56.3)	9.9 (55.9)
1 CCC	3.4 (20.9)	3.7 (21.8)	3.9 (22.2)	3.8 (21.4)	3.6 (21.2)	3.6 (20.3)	3.4 (19.5)	3.4 (18.9)

© 2025 Bayer ND et al. *JAMA Network Open*.

2 CCCs	1.3 (7.8)	1.4 (8.3)	1.7 (9.5)	1.9 (10.4)	1.9 (11.1)	2.0 (11.4)	2.0 (11.3)	2.0 (11.3)
≥3 CCCs	0.5 (3.3)	0.6 (3.7)	0.9 (4.9)	1.2 (6.8)	1.4 (8.5)	2.0 (11.4)	2.3 (12.8)	2.5 (13.8)
**Charges ($ billions)[4]**	**119.2**	**140.7**	**155.0**	**162.4**	**162.8**	**176.3**	**185.9**	**195.9**
No CCC	66.5 (55.8)	77.7 (55.3)	81.7 (52.7)	80.6 (49.7)	73.4 (45.1)	72.4 (41.1)	75.3 (40.5)	79.3 (40.5)
1 CCC	32.5 (27.3)	39.4 (28.0)	43.4 (28.0)	43.9 (27.0)	41.8 (25.7)	42.4 (24.0)	42.4 (22.8)	42.8 (21.9)
2 CCCs	13.9 (11.6)	16.3 (11.6)	20.0 (12.9)	22.9 (14.1)	25.4 (15.6)	28.5 (16.1)	29.5 (15.8)	30.4 (15.5)
≥3 CCCs	6.3 (5.3)	7.3 (5.2)	9.8 (6.3)	15.0 (9.2)	22.2 (13.6)	33.1 (18.8)	38.7 (20.8)	43.3 (22.1)

CCC = Complex Chronic Condition
[1] Shown are the retained numbers of hospital discharges and percentage of weighted hospital discharges for all births and non-births for ages 0-to-21 years with each of the three exclusions
[2] Identified with Major Diagnostic Category 14 from the Medicare Severity Diagnosis Related Groups (MS-DRG) system
[3] Identified with Kids' Inpatient Database elements "UNCBRTH" or "I10_UNCBRTH"
[4] Hospital charges were adjusted for inflation to 2022 U.S. dollars using the medical component of the Consumer Price Index.

© 2025 Bayer ND et al. *JAMA Network Open*.

eTable 2. National Estimates of Sociodemographic and Clinical Characteristics of Hospital Discharges for Children Aged 0 to 18 Years in the Kids' Inpatient Database 2000, 2003, 2006, 2009, 2012, 2016, 2019, and 2022.

Characteristic	Hospital Discharges		
	Overall [n (%)]	No CCC [n (%)]	≥ 1 CCC [n (%)]
**No. of Hospital Discharges**	26,342,497 (100)	21,033,576 (79.9)	5,308,921 (20.1)
**Age**			
<1 years	14,590,111 (55.4)	12,484,008 (59.4)	2,106,103 (39.7)
1-5 years	4,394,279 (16.7)	3,240,100 (15.4)	1,154,179 (21.7)
6-12 years	3,615,353 (13.7)	2,546,295 (12.1)	1,069,058 (20.1)
13-18 years	3,742,753 (14.2)	2,763,172 (13.1)	979,582 (18.5)
**Sex, male**	14,182,757 (54.1)	11,272,702 (53.9)	2,910,055 (54.9)
**Five Most Common Primary Diagnoses (by MDC)**			
Newborn Conditions During Perinatal Period	11,310,712 (42.9)	9,980,985 (47.5)	1,329,727 (25.0)
Respiratory System	3,653,818 (13.9)	3,052,735 (14.5)	601,083 (11.3)
Digestive System	1,882,210 (7.1)	1,481,565 (7.0)	400,645 (7.5)
Nervous System	1,283,432 (4.9)	734,165 (3.5)	549,267 (10.3)
Mental Diseases and Disorders	1,270,713 (4.8)	1,191,204 (5.7)	79,509 (1.5)

CCC = Complex Chronic Condition
MDC = Major Diagnostic Category

© 2025 Bayer ND et al. *JAMA Network Open*.
