# Supplementary material for: Trends in Hospital Resource Use for Children With Complex Chronic Conditions
Source: JAMA Netw Open. 2025 Dec 2;8(12):e2544686. doi: 10.1001/jamanetworkopen.2025.44686 (PMC12673417; doi:10.1001/jamanetworkopen.2025.44686)
Supplement: Supplement 2. — Data Sharing Statement [file jamanetwopen-e2544686-s002.pdf]

Data Sharing Statement

Bayer. Trends in Hospital Resource Use for Children With Complex Chronic Conditions. *JAMA Netw Open*. Published December 01, 2025. doi:10.1001/jamanetworkopen.2025.44686

Data
Data available: No

Additional Information
Explanation for why data not available: Data are not available for sharing by authors because the Kids' Inpatient Databases data are publicly available.
